# Supplementary material for: Decoding efficacy and resistance space at a drug binding site
Source: Nat Commun. 2026 Feb 4;17:2337. doi: 10.1038/s41467-026-69187-5 (PMC12979731; doi:10.1038/s41467-026-69187-5)
Supplement: Supplementary file 1 — Supplementary Information [file 41467_2026_69187_MOESM1_ESM.pdf]

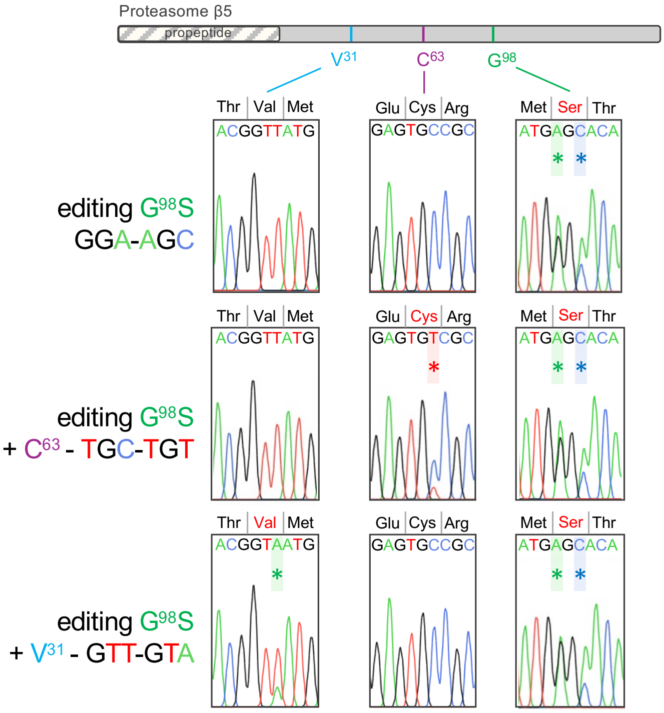

Supplementary Fig 1: Co-editing at adjacent sites following delivery of multiple ssODNs. The schematic representation of the *T. brucei β5* sequence highlights residues targeted for editing. Sequence traces show representative outcomes following single or dual ssODN delivery and drug-selection, with 10 nM DDD248. Edited nucleotides are marked by an asterisk.

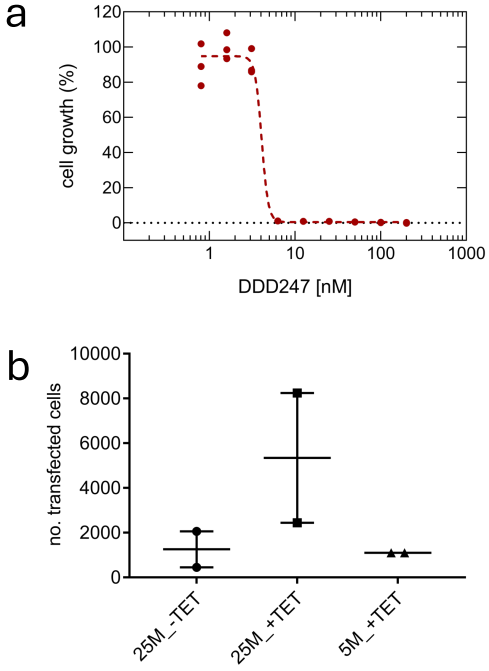

Supplementary Fig 2: Estimation of allele replacement frequency at the *β5* gene locus.

a The plot shows the *T. brucei* dose response curve for compound DDD247. Assays were carried out in triplicate and the EC_{50} was determined to be 4 nM. **b** An ssODN designed to introduce a GGA-AGC ($G^{98}S$) edit and selection with compound DDD247 6 h later were used to estimate allele replacement frequency at the β5 gene locus. We tested 25 million cells with or without induction of *MSH2* knockdown 24 h prior to transfection, and also 5 million cells with induction of *MSH2* knockdown. Transfected cells were serially diluted in 96-well plates immediately after adding the drug, and no. of transfected cells was estimated 5 days later. Considering 50 % survival following transfection, the assay yielded an allele replacement frequency of approx. 0.04 % following MSH2 knockdown

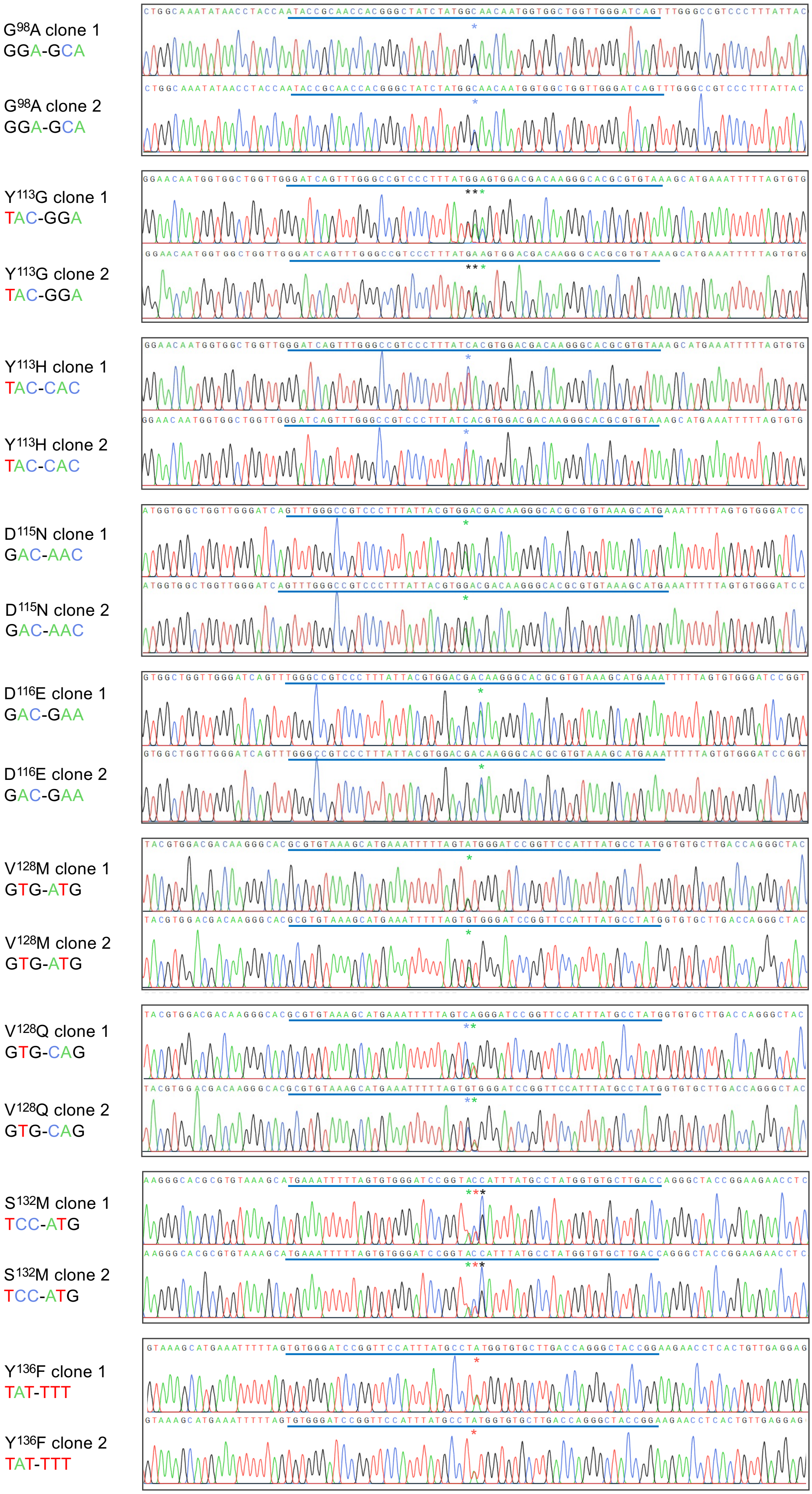

Supplementary Fig 3: Extended sequence traces for validation of edited clones. Sanger sequence traces for nine distinct mutations introduced into wild type *T. brucei*; two independent clones of each. Edited nucleotides are marked by an asterisk. The lines above each sequence track indicates the extent of each ssODN. Other details as in Fig. 3b.

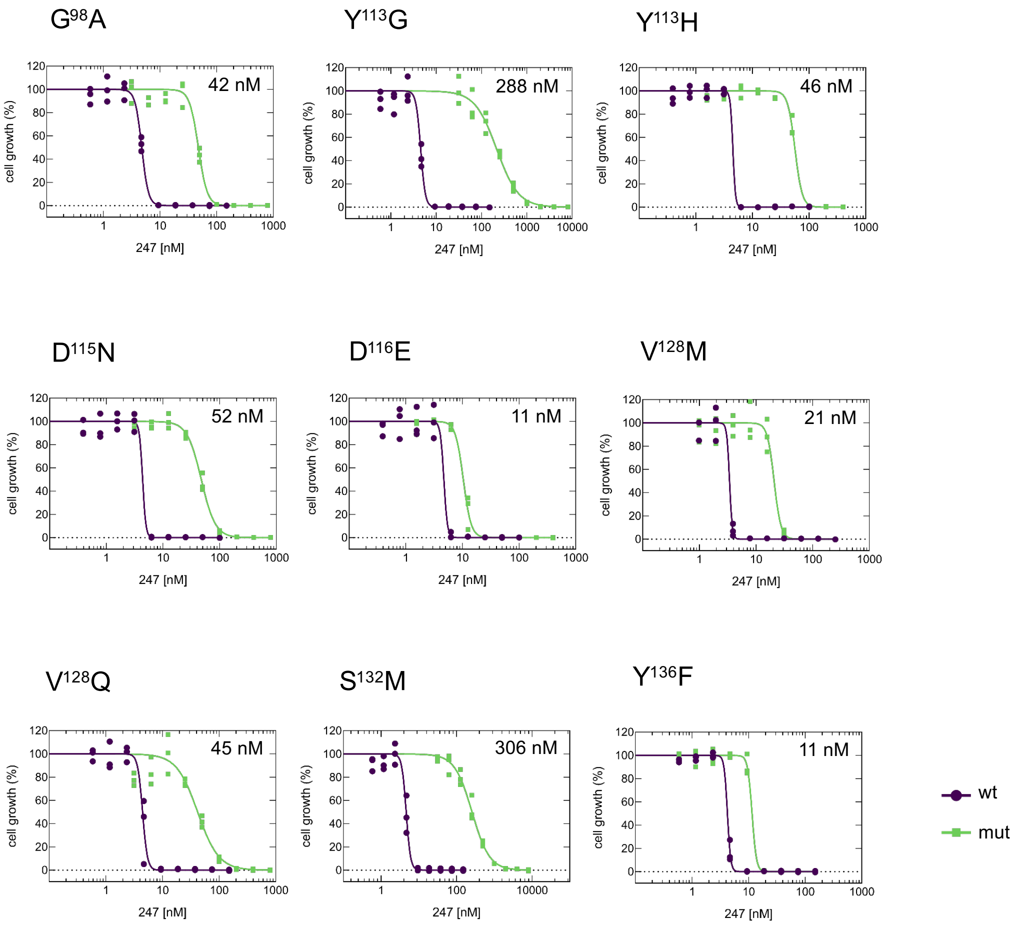

Supplementary Fig 4: Dose response curves for edited clones.

Further dose response curves for the panel of edited and drug-resistant mutants; second biological replicate for each edit. Other details as in Fig. 3c.

a

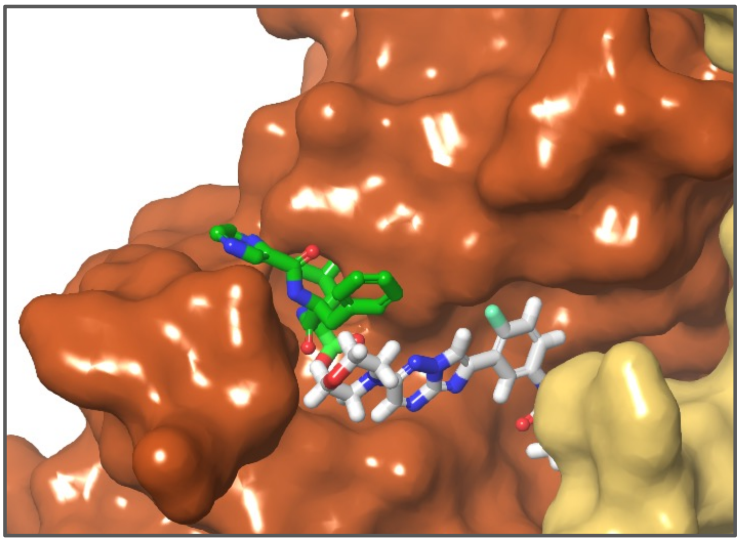

b

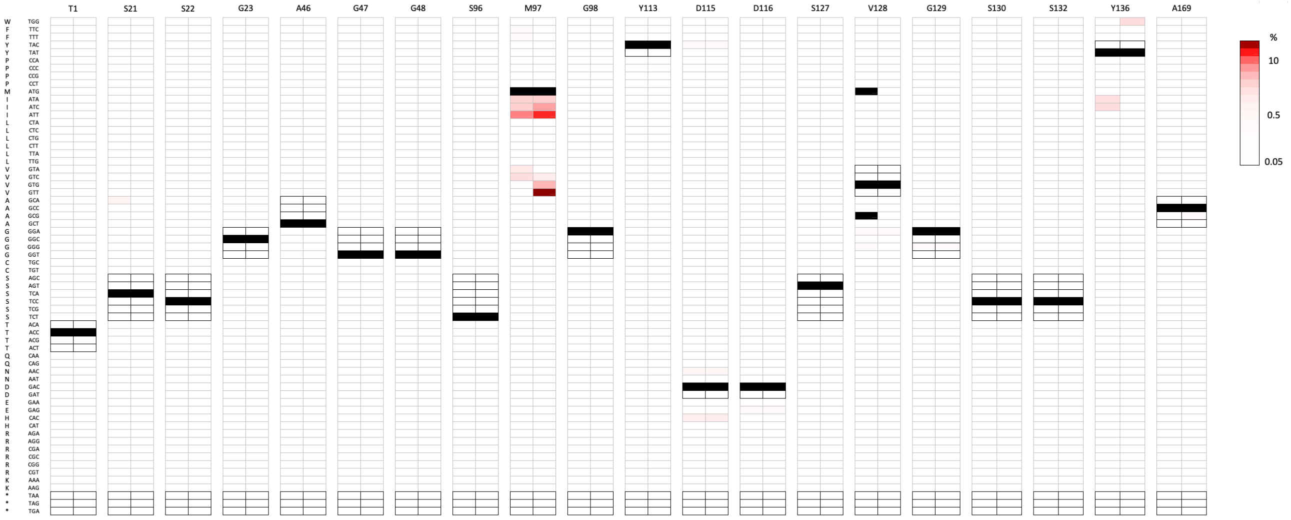

Supplementary Fig 5: MOT-library profiling with bortezomib.

a Docking model for DDD247 (grey) and bortezomib (green) with the *T. brucei* proteasome, indicating distinct, but adjacent binding sites. **b** Two MOT-libraries were selected with bortezomib (4nM) in parallel. All sixty-four possible codon variant scores for all twenty targeted sites in the proteasome *β5* subunit are represented as a heat-map. Unedited codons are indicated (black), while synonymous codons and stop-codons are highlighted with darker framed outlines.

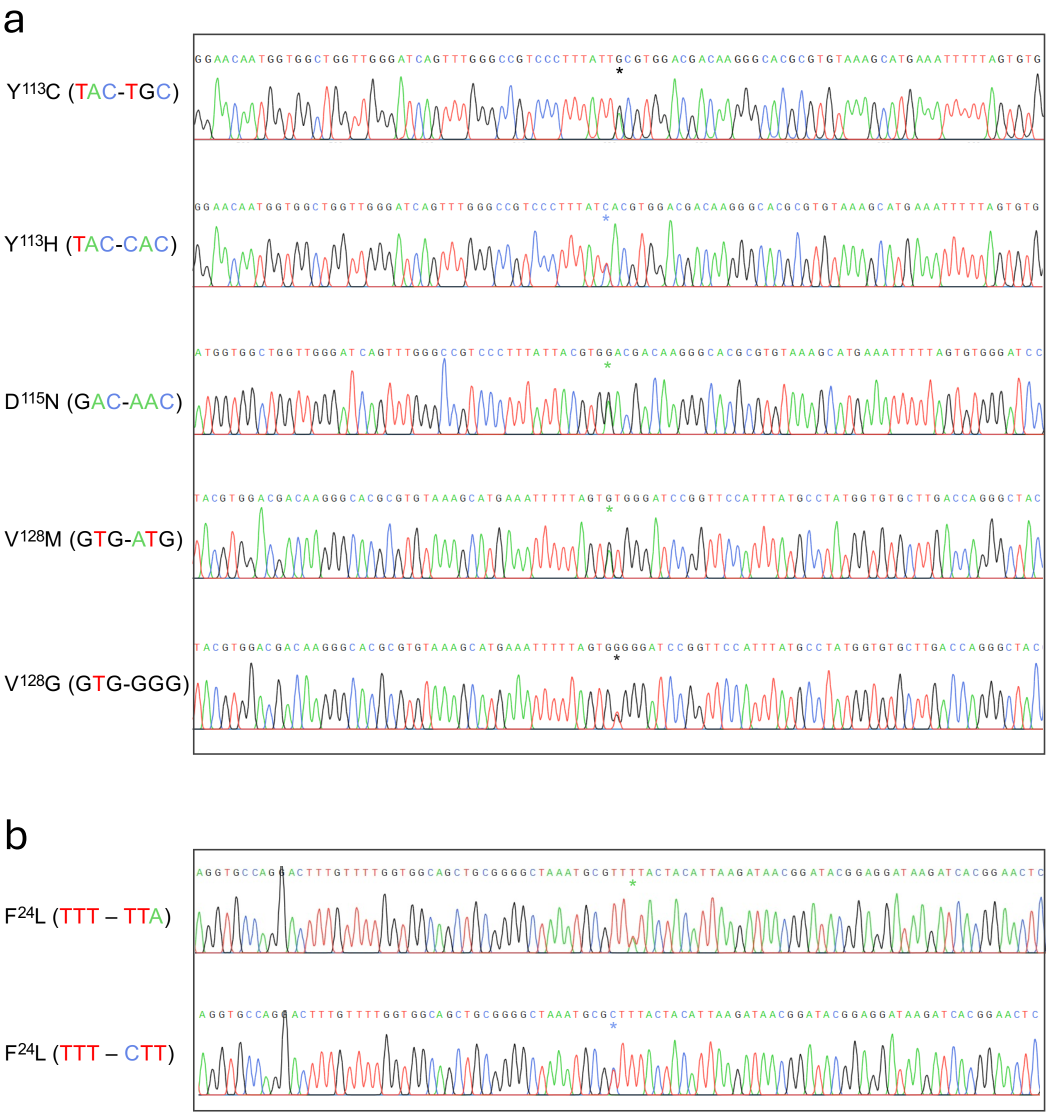

Supplementary Fig 6: Extended sequence traces for spontaneous DDD247-resistant mutants.

a Sanger sequencing traces for spontaneous mutations in the proteasome *β5* subunit gene following transient *MSH2* knockdown and associated with compound DDD247 resistance; all heterozygous single nucleotide variants. Mutated bases are marked by an asterisk. **b** As in a but for the proteasome *β4* subunit gene. Other details as in Fig 5c.

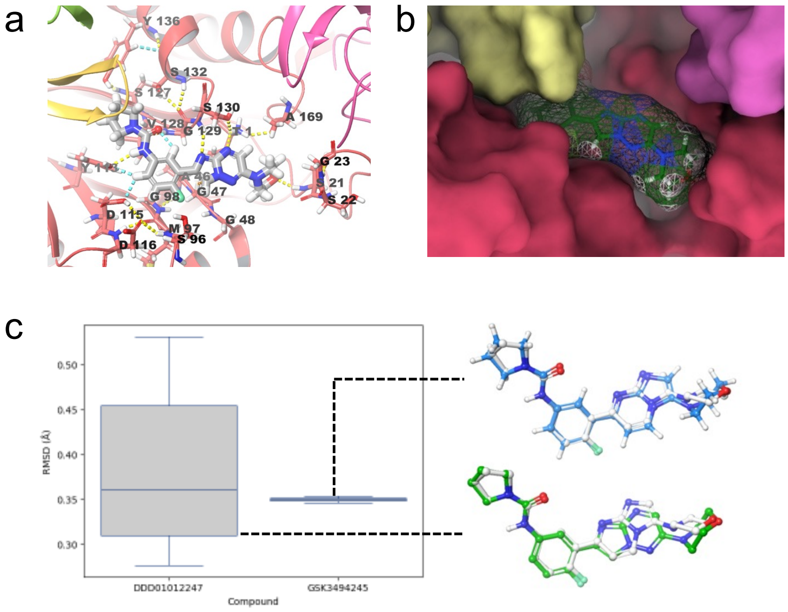

Supplementary Fig. 7: Assessment of the *T. brucei* proteasome homology model.

a Docking model for the T. brucei proteasome and compound DDD247, indicating sites surrounded the binding pocket. **b** DDD247 sits within a well-formed cavity, primarily interacting with the β5 subunit (red). **c** RMSD values derived from comparisons of compound DDD247 (*T. brucei*) v GSK245 (pdb-Id:6qm7) and GSK245 (*T. brucei*) v GSK245 (pdb-Id:6qm7); in both cases, the reference is shown in grey.

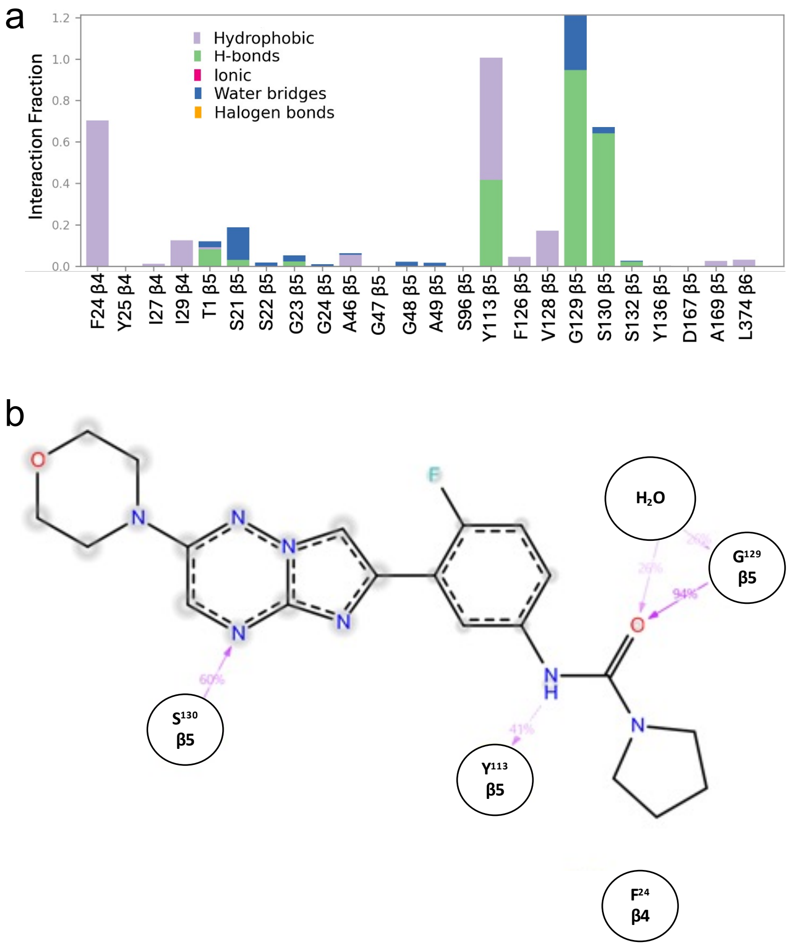

Supplementary Fig. 8: Summary of molecular interactions identified using molecular dynamics simulation.

a The plot indicates interaction fraction and interaction class between the DDD247 ligand and subunits in the *T. brucei* proteasome homology model. **b** Percentage of major interactions along the simulation. Gray diffuse rings indicate solvent-exposed residues.

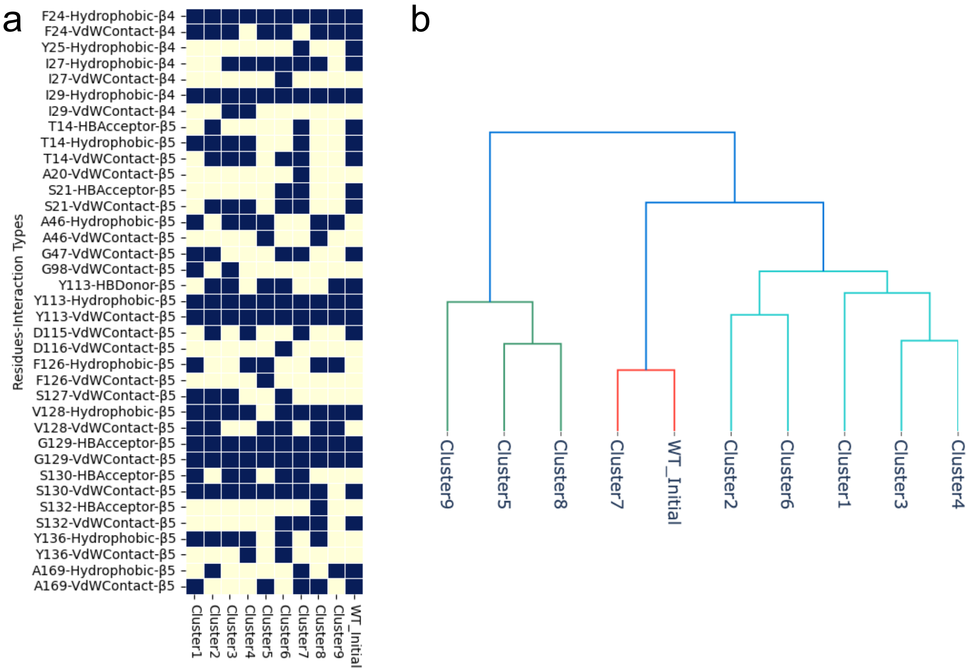

Supplementary Fig. 9: Assessment of the *T. brucei* proteasome molecular dynamics simulation.

A Interaction fingerprints (ProLIF) of each representative snapshot from clustering; dark blue indicates interactions with DDD247. **b** Fingerprint Tanimoto similarity tree for representative snapshots from each cluster. Cluster Set 1 (2,3,6,WT_initial) and cluster 8 were selected for further analysis; cluster 8 specifically to derive data for β5 subunit S^{132} mutations.

a

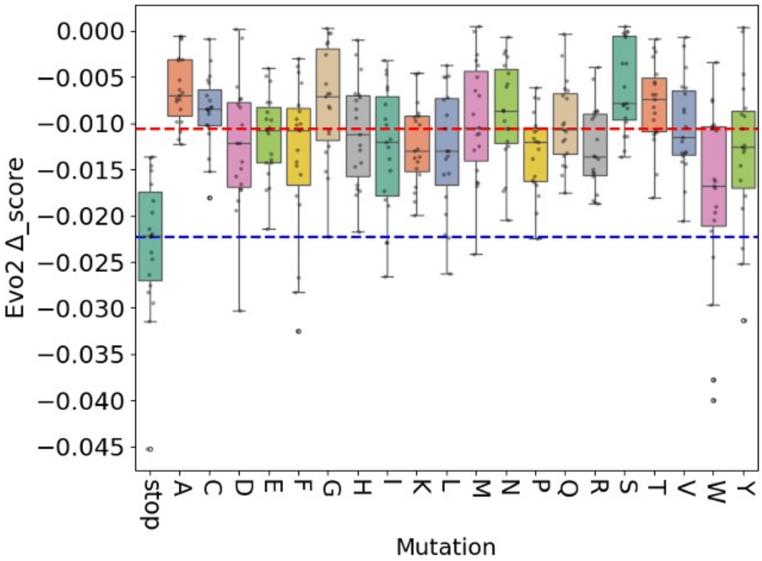

b

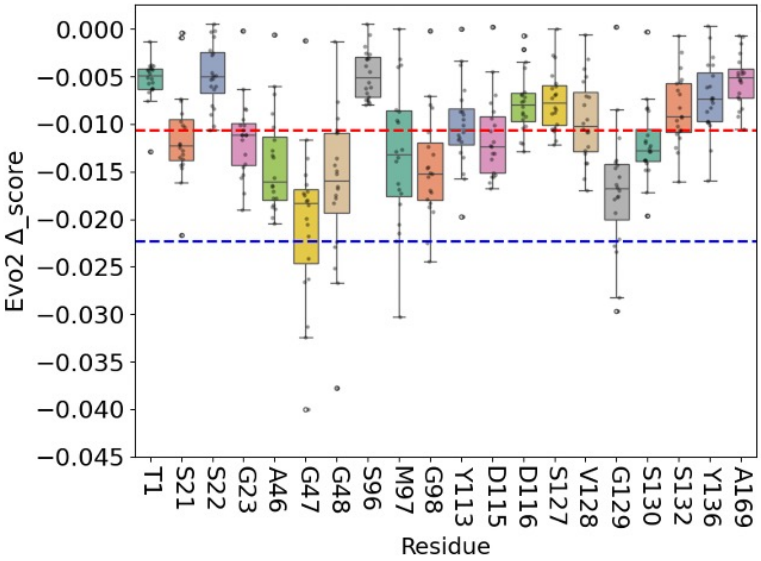

Supplementary Fig. 10: Functional predictions using Evo 2

a The boxplot shows Evo 2 predictions for mutations yielding each possible amino acid, across the twenty proteasome β5 subunit residues surveyed by MOT-profiling. The average Evo2 Δ_scores for codons encoding the same amino at each site are shown. The thresholds for predicting relative loss-of-function were selected based on the average predictions for stop codons (-0,0223, blue line, maximum loss-of-function) and average predictions for other codons (-0.0106, red line, no loss-of-function); values between these thresholds were used to apply a 'loss-of-function' penalty to our affinity predictions. Boxes indicate the interquartile range (IQR), and the whiskers show the range of values within 1.5×IQR. **b** The boxplot shows Evo 2 predictions for mutations yielding all possible amino acid changes at the twenty proteasome β5 subunit residues surveyed by MOT-profiling. Other details as in a.
